# Minimal invasive treatment of life-threatening bleeding caused by cardiopulmonary resuscitation-associated liver injury: a case report

**DOI:** 10.1186/s13256-016-0926-3

**Published:** 2016-05-29

**Authors:** Pål Aksel Næss, Kristian Engeseth, Ole Grøtta, Geir Øystein Andersen, Christine Gaarder

**Affiliations:** 1 Department of Traumatology, Oslo University Hospital Ullevål, Nydalen Postbox 4956, N-0424 Oslo, Norway; 2 Department of Gastrointestinal and Pediatric Surgery, Oslo University Hospital Ullevål, Oslo, Norway; 3 Department of Cardiology, Oslo University Hospital Ullevål, Oslo, Norway; 4 Department of Radiology and Nuclear Medicine, Oslo University Hospital Ullevål, Oslo, Norway

**Keywords:** Cardiopulmonary resuscitation, Liver injury, Angiographic embolization, Case report

## Abstract

**Background:**

Life-threatening bleeding caused by liver injury due to chest compressions is a rare complication in otherwise successful cardiopulmonary resuscitation. Surgical intervention has been suggested to achieve bleeding control; however, reported mortality is high. In this report, we present a brief literature review and a case report in which use of a less invasive strategy was followed by an uneventful recovery.

**Case presentation:**

A 37-year-old white woman was admitted after out-of-hospital cardiac arrest. Bystander cardiopulmonary resuscitation was immediately performed, followed by advanced cardiopulmonary resuscitation that included tracheal intubation, mechanical chest compressions, and external defibrillation with return of spontaneous circulation. Upon hospital admission, the patient’s blood pressure was 94/45 mmHg and her heart rate was 110 beats per minute. Her electrocardiogram showed no signs of ST-segment elevations or Q-wave development. Coronary angiography revealed a proximal thrombotic occlusion of the left anterior descending coronary artery. Successful recanalization, after thrombus aspiration and balloon dilation followed by stent implant, was verified with normalized anterograde flow. Immediately after the patient’s arrival in the intensive cardiac care unit, a drop in her blood pressure to 60/30 mmHg and a hemoglobin concentration of 4.5 g/dl were noticed. Transfusion was started, and bedside abdominal ultrasound examination revealed free intraperitoneal fluid. Computed tomography of the abdomen revealed liver injury with active extravasation from the cranial surface of the right lobe and a massive hemoperitoneum. The patient was coagulopathic and acidotic with a body temperature of 33.5 °C. A minimally invasive treatment strategy, including angiography and selective trans-catheter arterial embolization, were performed in combination with percutaneous evacuation of 4.5 L of intraperitoneal blood. After completion of these procedures, the patient was hemodynamically stable. She was weaned off mechanical ventilation 2 days later and made an uneventful recovery. She was discharged to a local hospital on day 13 without neurological disability.

**Conclusions:**

Although rare, bleeding caused by liver injury due to chest compressions can be life-threatening after successful cardiopulmonary resuscitation. Reported mortality is high after surgical intervention, and patients may benefit from less invasive treatment strategies such as those presented in this case report.

## Background

Although rare, bleeding caused by liver injury due to chest compressions can be life-threatening after successful cardiopulmonary resuscitation (CPR) [1, 2]. Surgical intervention has been advocated to obtain bleeding control [1]. However, reported mortality is high, and patients may benefit from less invasive treatment strategies such as those presented in this case report.

## Case presentation

### Case report

Our patient was a 37-year-old, obese, previously healthy white woman. She was a smoker with no family history of ischemic heart disease. Her recent history involved 3 weeks of respiratory tract infection treated with antibiotics and bronchodilators.

She was admitted to our hospital after experiencing a witnessed out-of-hospital cardiac arrest. Bystander CPR was immediately performed for 7 minutes, followed by advanced CPR that included tracheal intubation and mechanical chest compressions provided by the physician-led ambulance team. Her electrocardiogram (ECG) revealed ventricular fibrillation, and she underwent external defibrillation with return of spontaneous circulation. Upon hospital admission, the patient’s blood pressure (BP) was 94/45 mmHg and her heart rate was 110 beats per minute. Her ECG initially showed atrial fibrillation with spontaneous conversion to sinus rhythm and no signs of ST-segment elevations or Q-wave development. Computed tomography (CT) scans of her head and chest were reported normal.

Coronary angiography revealed a proximal thrombotic occlusion of the left anterior descending (LAD) coronary artery. Successful recanalization of the LAD coronary artery after catheter-based thrombus aspiration, and balloon dilation followed by stent implantation, was verified with normalized anterograde flow (Thrombolysis in Myocardial Infarction grade flow score 3), and an eptifibatide infusion was instituted.

**Fig. 1:**
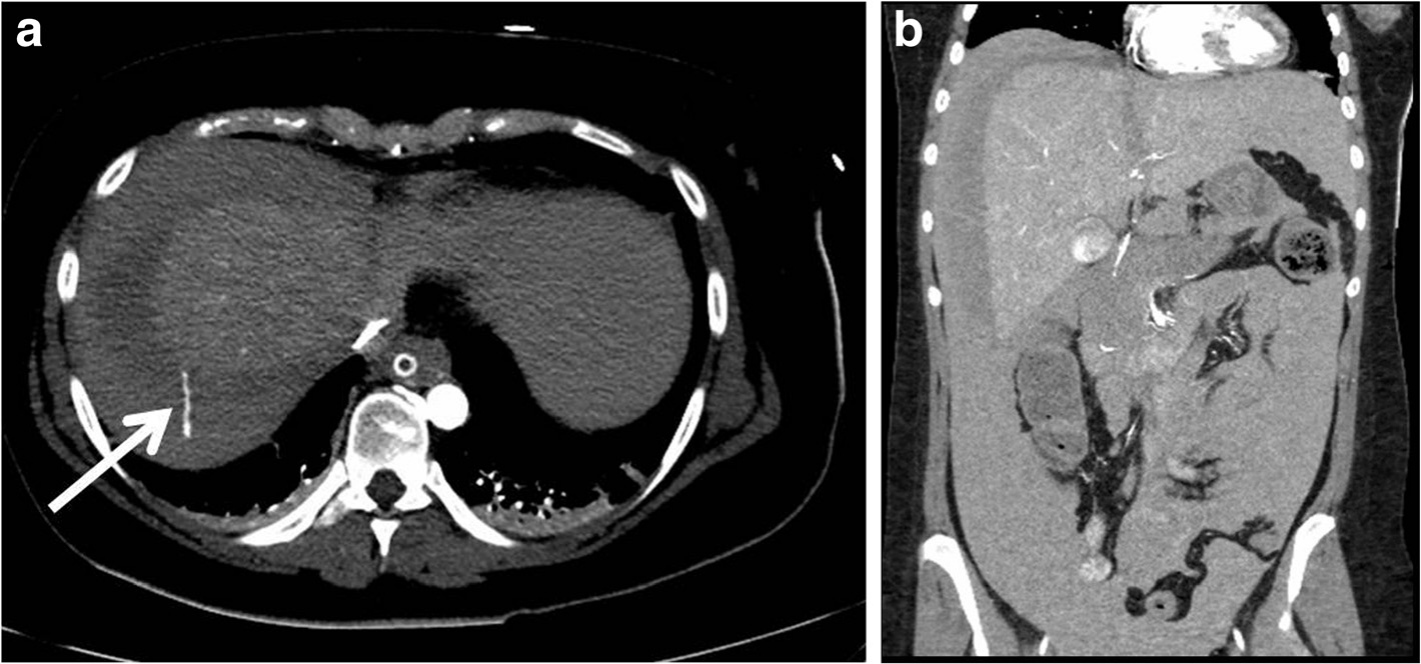
Axial (**a**) and coronal (**b**) computed tomographic images of the abdomen revealing active contrast extravasation from the liver (*arrow*) and a massive hemoperitoneum

Immediately after the patient’s arrival in the intensive cardiac care unit (ICCU), a drop in her BP to 60/30 mmHg and a hemoglobin concentration of 4.5 g/dl were noticed. Transfusion was started, the patient’s abdomen was distended, and bedside abdominal ultrasound (US) examination revealed free intraperitoneal fluid. Abdominal CT (Fig. 1) performed after initial stabilization revealed liver injury with active extravasation from the cranial surface of the right lobe and a massive hemoperitoneum.

**Fig. 2:**
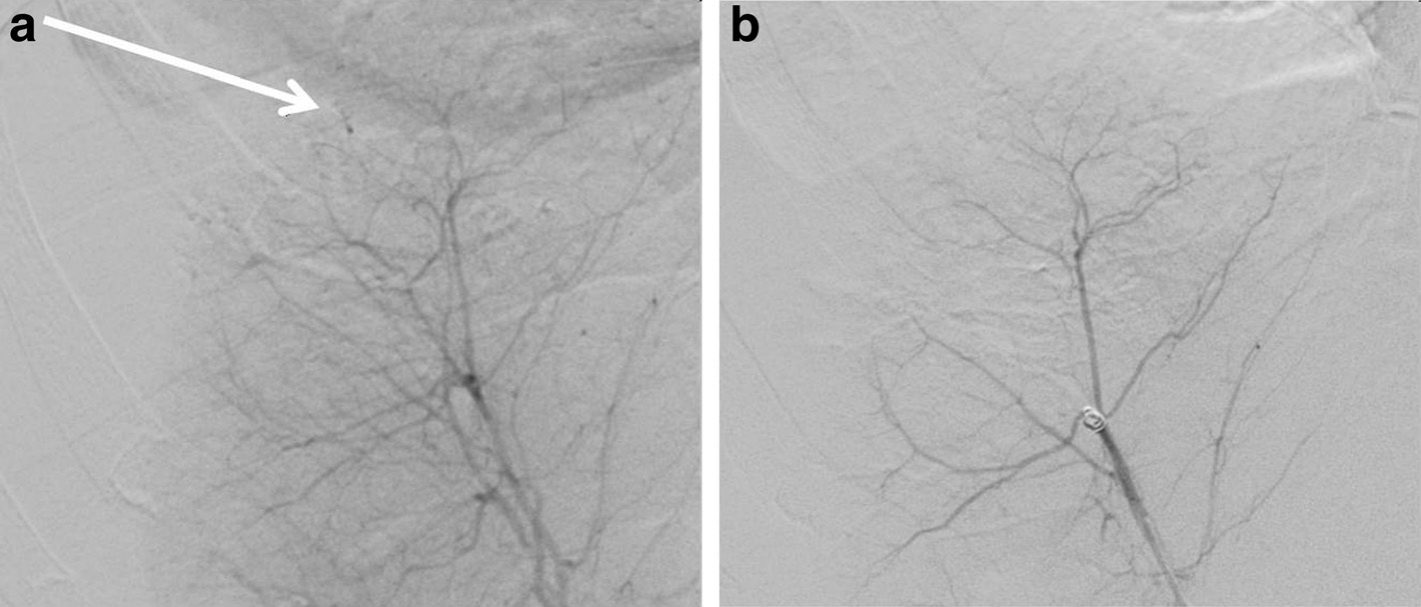
Digital subtraction angiography confirming extravasation (*arrow*) from the liver surface (**a**) and cease of extravasation after selective embolization with Gelfoam (Pfizer, New York, NY, USA) and a coil (**b**)

**Fig. 3:**
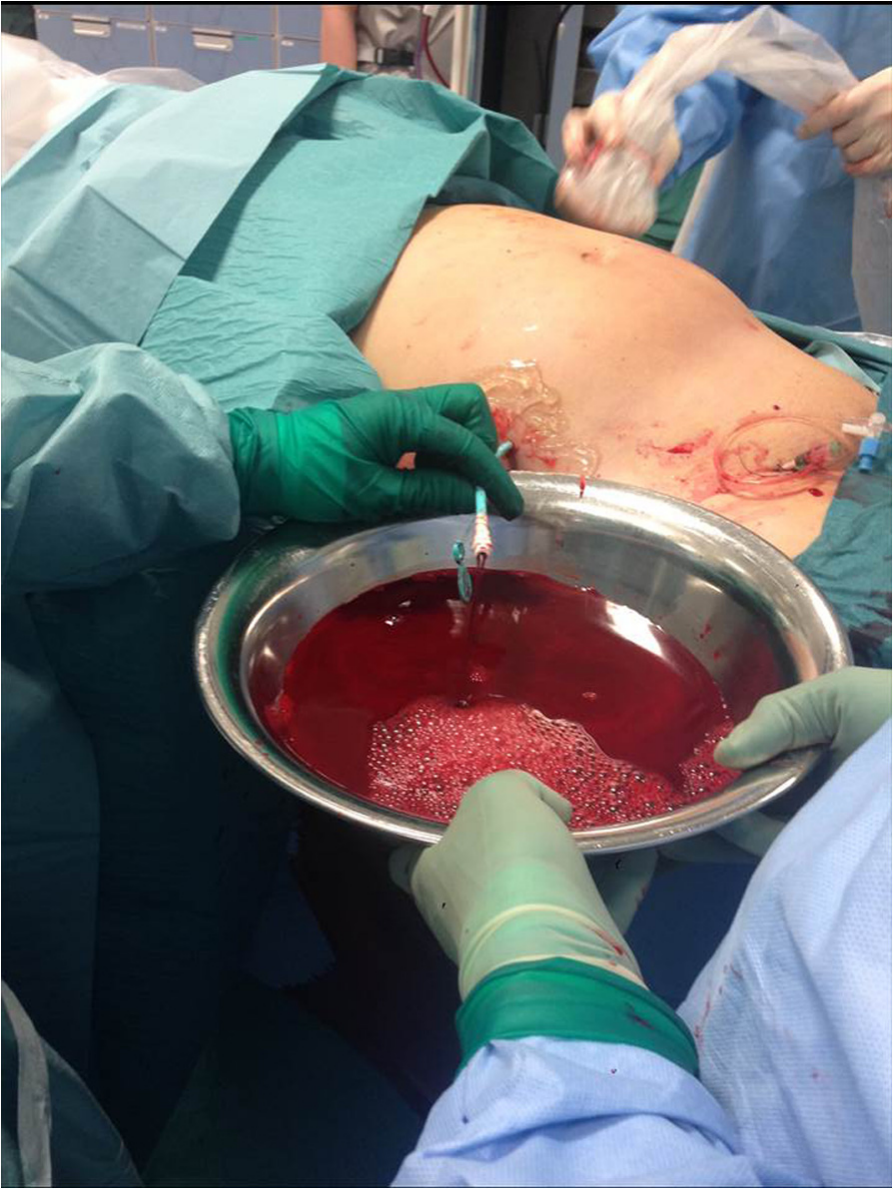
Rapid evacuation of massive hemoperitoneum through a 16-French pigtail catheter inserted under ultrasound guidance

At that point, the patient was coagulopathic with a body temperature of 33.5 °C. Arterial blood gas analysis revealed a pH of 7.15 and a lactate concentration of 7.4 mmol/L. Angiography and selective transcatheter arterial embolization (Fig. 2) were successfully performed in combination with evacuation of 4.5 L of intraperitoneal blood through a 16-French pigtail catheter inserted into the right flank under US guidance (Fig. 3).

After completion of these procedures, the patient was transfused with a total of 9 U of packed red blood cells, 8 U of plasma, and 2 U of platelets (pooled from five donors each) and was hemodynamically stable. She was weaned off mechanical ventilation 2 days later and thereafter made an uneventful recovery. She was discharged to a local hospital on day 13 without neurological disability.

## Discussion

Chest compressions performed as part of immediate CPR play a fundamental role in survival from cardiac arrest; however, they are frequently associated with complications of varying severity [1, 3, 4]. The most commonly reported CPR-related injuries are rib and sternal fractures that do not usually contribute to a patient’s mortality [1, 3, 5, 6].

Liver injuries are infrequent complications of CPR, with a reported incidence of approximately 0.6 % [1, 2, 7]. A high degree of awareness is required to make an early diagnosis of life-threatening bleeding caused by a liver injury after initial successful CPR [2, 3]. Most resuscitated patients have a reduced level of consciousness and therefore are unable to explain their symptoms [5]. Moreover, bleeding from hepatic injuries may progress slowly, with signs of hypovolemia not developing until several hours after CPR, mimicking the hemodynamic instability due to the myocardial dysfunction associated with the post-cardiac arrest syndrome [1, 3, 5, 8].

The observed drops in BP and hemoglobin concentration in our patient strongly indicated massive bleeding. Both US and CT have been recommended as sensitive methods for the detection of intraabdominal bleeding in this category of patients [2, 3, 5]. Abdominal US performed in the ICCU showed large amounts of intraabdominal free fluid, and subsequent CT verified ongoing bleeding from the right lobe of the liver (Fig. 1).

On the basis of their experience with the largest series of patients reported, Meron *et al*. [1] recommended laparotomy as the method of choice for stopping life-threatening bleeding caused by CPR-related liver injuries. Nevertheless, in spite of successful surgical intervention, only one of their six patients survived. Laparotomy is hazardous in a coagulopathic, hypothermic, and acidotic patient. In our patient, immediate control of life-threatening bleeding from the liver and evacuation of intraperitoneal blood to avoid fulminant abdominal compartment syndrome were crucial. Angiography and selective transcatheter arterial embolization, combined with laparocentesis, were considered the least risky options and were successfully performed. These procedures were followed by an uneventful recovery.

## Conclusions

The benefit of chest compressions as part of CPR in patients with cardiac arrest overshadows the rare complication of life-threatening bleeding caused by liver injury. However, the probability of this complication should be considered when a patient with preceding CPR shows discreet clinical signs of bleeding. Surgical intervention has been suggested to achieve bleeding control; however, reported mortality is high. A minimally invasive treatment strategy that includes angiographic embolization and percutaneous evacuation of intraabdominal blood may favorably affect outcome in these patients.

## Consent

Written informed consent was obtained from the patient for publication of this case report and any accompanying images. A copy of the written consent is available for review by the Editor-in-Chief of this journal.
